# The Development of Associate Learning in School Age Children

**DOI:** 10.1371/journal.pone.0101750

**Published:** 2014-07-11

**Authors:** Brian T. Harel, Robert H. Pietrzak, Peter J. Snyder, Elizabeth Thomas, Linda C. Mayes, Paul Maruff

**Affiliations:** 1 CogState, Ltd., New Haven, Connecticut, United States of America; 2 Department of Psychiatry, Yale University School of Medicine, New Haven, Connecticut, United States of America; 3 Department of Neurology, Alpert Medical School of Brown University, Providence, Rhode Island, United States of America; 4 School of Behavioural Science, Medicine, Dentistry and Health Sciences, University of Melbourne, Melbourne, Victoria, Australia; 5 Yale Child Study Center, Yale University School of Medicine, New Haven, Connecticut, United States of America; 6 Paul Maruff, CogState Ltd., Melbourne, Victoria, Australia; Utrecht University, Netherlands

## Abstract

Associate learning is fundamental to the acquisition of knowledge and plays a critical role in the everyday functioning of the developing child, though the developmental course is still unclear. This study investigated the development of visual associate learning in 125 school age children using the Continuous Paired Associate Learning task. As hypothesized, younger children made more errors than older children across all memory loads and evidenced decreased learning efficiency as memory load increased. Results suggest that age-related differences in performance largely reflect continued development of executive function in the context of relatively developed memory processes.

## Introduction

Associate learning is the process by which different aspects of information are encoded into memory such that later exposure to one aspect of that same information elicits recall of the other [1]–[4]. In neuropsychology, associate learning is often measured by having individuals learn multiple pairs of information, such as, nouns and adjectives, faces and names or objects and colors. Provided the number of pairs to be learned exceeds working memory capacity (e.g., one or two pairs), associate learning abilities are defined by the extent to which individuals who have learned those pairs can later recall one of the pair after exposure to the other [2], [5]–[11]. Because associate learning is important for optimal adaptive behavior in both educational and general life contexts, it is important to understand its development in children [3], [4], [12]–[14]. Furthermore, identification of impairment in associate learning in children can assist with the identification or diagnosis of brain disorders [15]–[18].

Developmental studies show associate learning improves from childhood through adolescence into young adulthood [3], [19]–[23]. However, as most of these studies have measured the ability to form associations between verbal stimuli [19], [23] or easily verbalized visual stimuli (e.g. pictures of objects), their results might reflect the maturation of language as much as memory [20]–[22], [24]. Developmental neuropsychologists emphasize that in order to understand cognitive development, it is important to control the influence of language ability in children. This is because language improves with age and can substantially affect performance on cognitive tasks of higher cognitive functions which themselves are not primarily linguistic in nature, such as executive functions or associate learning [25], [26]. This has been demonstrated in verbal paired associate learning where performance on verbal associate learning tasks has been shown to correlate with reading ability [12], [27]–[29] while performance on a visual paired associate learning task using abstract, and therefore difficult to verbalize, patterns did not [12].

Another important issue in understanding the development of associate learning arises from current adult neuropsychological models that contend that, in addition to memory encoding and retrieval processes, the ability to learn associations is dependent on executive functions, such as organization, search strategy, and response monitoring [13], [30], [31]. This two-component framework of associate learning is based on data from brain lesion [8]–[10], [32] and neuroimaging [13], [30], [31], [33] studies. The specific role of executive functions in associate learning is dependent upon the characteristics of the task. In associate learning tasks where learning occurs incrementally through trial and error with repeated exposure to correct and incorrect responses, as is the case in our associate learning task described below, successful performance is strongly dependent upon executive functions [32], [34]. Specifically, performance requires the subject to consider an increasing number of prior responses, determine which were correct and which were incorrect, and then use that information to guide the current response [32], [34].

As executive functions related to strategy and problem solving ability also develop through childhood and into adolescence, it remains possible that age-related improvement on associate learning tasks might also reflect, at least in part, development of these aspects of executive function [23], [35]. Working memory capacity also improves through childhood [36]–[40]. Therefore, age-related improvement in associate learning might also reflect an increased efficiency in encoding of paired information due to the maturation of working memory capacity [41]–[43].

No studies of associate learning have sought to understand how this theoretical framework contributes to the development of visual associate learning. Shing and colleagues (2008) investigated the development of memory and executive components of verbal associate learning by comparing the ability of children (aged 10–12 years), teenagers (aged 13–15 years) and young adults (aged 20–25 years) to learn word pairs under different conditions. The memory component of associate learning was manipulated by varying the associative strength of word pairs while the executive component was manipulated by varying the degree to which the study instructions emphasized strategic encoding. Compared to adults, children's performance was poorer on the associate learning tasks though this reflected limitations in strategy use and not any limitations in forming associations. These results are consistent with developmental neuropsychological studies which observe performance on simple memory tasks reach adult levels by early to middle childhood [44]–[46], whereas performance on more difficult tasks of executive functions do not reach adult levels until late childhood [45], [47]. Unfortunately, as Shing and colleagues used a verbal associate learning paradigm, the absence of any limits in forming associations might reflect mature language as opposed to memory processes. Although it is well-known that memory, executive functions, and working memory capacity become more efficient at differential rates as children age, this knowledge is based on performance on different tasks or modifications to the same task [23]. To the best of our knowledge, no study has investigated how maturation of these different processes are integrated within the same task in order to understand performance on a complex learning paradigm. Recent work has highlighted the limitations of this approach [48]. For instance, it has been argued that the substantial variability of working memory capacity estimates across tasks and domains within the same age groups is due to the fact that performance on a given working memory task is the consequence of the integrative actions of multiple cognitive processes [48]. As such, in order to examine how different cognitive processes develop in relation to each other as children age, it is important to do so within the same task.

**Figure 1:**
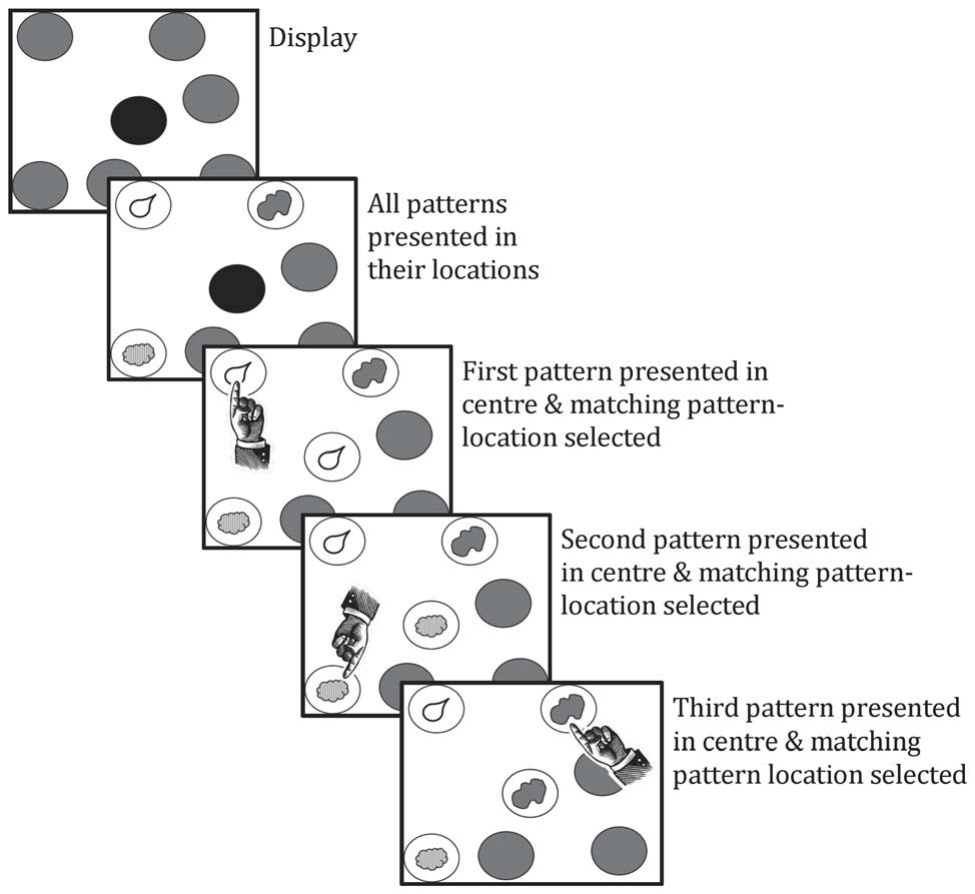
CPAL task exposure phase.

**Figure 2:**
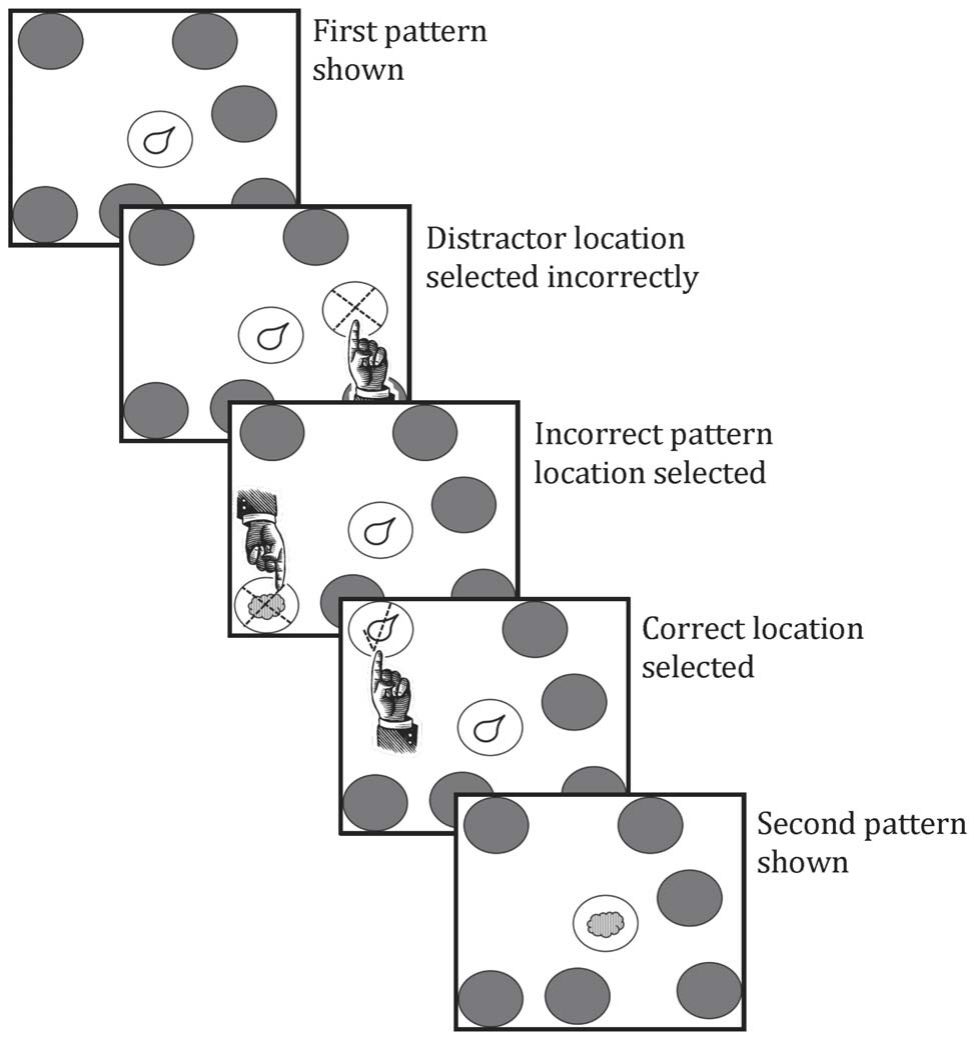
CPAL task learning phase.

Recently, a version of the visual paired associate learning task was developed (Continuous Paired Associate Learning task; CPAL) that required individuals to learn sets of associations between locations and abstract, difficult to verbalize patterns. In an exposure phase (see Figure 1) all patterns are shown in their locations and one pattern at a time appears in the center of the screen. Individuals must match the pattern at the location in the periphery with that shown in the center (see Figure 2). Once matched, patterns in the periphery are occluded. In the learning phase, one of the patterns appears in the center of the screen and the individual is required to indicate the peripheral location containing that pattern. The search for the matching peripheral location continues until the correct location is found while the pattern in the central location remains visible. During the search, error feedback is provided after incorrect choices (see Table 1). Errors made while searching novel locations are considered to reflect consolidation of pattern-location associations into memory (memory processes; [49]–[51]. In contrast, errors made while searching locations already associated with a different pattern or already found to be incorrect are considered to reflect impairment in executive processes such as search strategy and working memory [49]–[51].

**Table 1:** Classification of Errors That Can Be Made on the Continuous Paired Associate Learning Task.

Error subtype	Component cognitive process	Description
Exploratory*	Visual learning	Where a location that has not been searched previously or associated correctly with a pattern on a previous search is selected
Distractor-search*	Visual learning	Where a location at which a target has never been shown is searched
Between-search∧	Executive function (strategic search)	Where a location that has already been associated correctly with a pattern in a previous search is selected
Within-search∧	Executive function (working memory)	Where on the current search, a location that has already been searched is selected a second time after at least one other location has been searched
Perseverative	Executive function (response monitoring)	Where the same incorrect location is searched with consecutive selections

*Note.*

*Exploratory and Distractor-search errors are aggregated to calculate the memory errors score.

∧ *Between-search and Within-search errors are aggregated to calculate the executive errors score.*

There is now evidence that the contribution of memory and executive components to performance on the CPAL can be separated when the to-be-learned information exceeds working memory capacity. First, in individuals with a subtle amnesia due to Alzheimer's disease (i.e., mild cognitive impairment (MCI) with abnormal amyloid levels) poor associate learning ability was due to difficulty in learning associations while executive components remained relatively normal [49], [52], [53]. In contrast, performance on the CPAL in adults with subtle amnesia not related to AD (i.e., MCI without abnormal amyloid levels) was due mainly to impairment in both the memory and executive components of associate learning [52]. In healthy young adults, treatment with sedative drugs such as benzodiazepines or anticholinergics reliably impairs associate learning [51], [54]–[57]. However, this was due to greater impairment in the memory component of associate learning rather than the executive component [51]. Conversely, while treatment with the muscarinic agonist scopolamine also impaired associate learning in young adults, this impairment was characterized by greater difficulty in the executive component of associate learning than with the memory component [56]. Taken together, the dissociations between memory and executive components of associate learning observed in the clinical and pharmacological studies suggest strongly that these two aspects of associate learning can be assessed using the CPAL. Thus, the use of non-verbal stimuli, variable numbers of to-be-learned associations and the ability to derive valid measures of the memory and executive components of associate learning make the CPAL potentially useful for understanding how multiple cognitive operations are integrated to give rise to visual associate learning in children.

The aim of this study was to investigate the development of visual associate learning, and the contributions of memory and executive functions to that development, in children under conditions of increasing memory load using the CPAL. The first hypothesis was that visual associate learning would become less efficient as memory load increased. The second hypothesis was that under conditions where working memory capacity was exceeded, visual associate learning would improve with increasing age. We then examined the extent to which age and increasing memory load influenced memory and executive components of visual associate learning.

## Method

### Ethics Statement

Consent forms were sent home to either caretakers, guardians or parents of the children and informed consent was obtained in writing. This study has been approved by the Human Ethics Committee at the University of Melbourne.

### Participants

One hundred and twenty six children, aged five to 10 years, participated in the study, however one child in the 5–6 year old group was unable to complete the CPAL and, as such, was excluded from the sample. Children were selected from the first to fifth years at elementary schools in a large regional city in Australia. Parents of children were sent information on the study together with consent forms and a questionnaire requesting demographic and developmental information. Children with a history of physical, sensory or cognitive impairment, or those enrolled in special education classes were excluded. Children receiving central nervous system active medication were also excluded. The sample consisted of 30 children aged five and six years (15 males and 15 females, mean age 66.2 months, SD = 6.9 months), 49 children aged seven and eight years (32 males and 17 females, mean age 91.2 months, SD = 7.6 months) and 46 children aged nine and 10 year (24 males and 22 females, mean age 115.2 months, SD = 7.9 months). All children spoke English as their first language.

### The Continuous Paired Associate Learning Task (CPAL)

The CPAL has been described in detail elsewhere [49] and is shown graphically in Figures 1 and 2. Briefly, the display for the CPAL consists of a single location appearing surrounded by 3 to 12 peripheral locations; depending on the number associations to be learned. With each CPAL administration, peripheral locations were marked at a random sample of 80 possible but otherwise unmarked positions on the display with the vector distance from the center of the display to the center of all peripheral locations summed to zero (i.e., the central location appeared to be the center of gravity of the peripheral locations). Stimuli for the CPAL were drawn at random from a library of 30 patterns, each of which was a solid, single colored amoeboid shape and which differed from all the others according to the combination of color and shape. In the current study, associate memory load was varied by testing performance for 2, 4, 6, 8 and 10 pattern-location associations. The CPAL proceeded using an exposure phase and a learning phase.

#### Exposure phase

The exposure phase of the CPAL begins with all of the to-be-remembered pattern-location associations for a given memory load presented on the display simultaneously. After a five second delay, a pattern is presented in the central location indicating that the participant should touch the location in the periphery that contains the same pattern. The pattern in the center remains present until the correct location in the periphery is selected. When the peripheral location containing the correct pattern is touched the pattern is occluded and a second pattern is shown in the central location. This process continues until each of the patterns in their peripheral locations have been touched and then occluded.

#### Learning phase

The learning phase of the CPAL begins with the same task display as that presented during the exposure phase except that now all peripheral locations are occluded. One of the patterns, presented in the exposure phase, is then shown in the center location indicating that the participant should select the peripheral location where that pattern had been presented initially. If the correct location is selected, the occlusion is removed to show the pattern in the correct location and a check mark is drawn. If the response is incorrect a “beep” sound occurs and the occlusion is removed from the location selected to show either that the location contains a different pattern, or is empty and then the occlusion is replaced. A cross indicating the selection is incorrect is also superimposed on the incorrect location. This process continues until the correct location for the pattern presented in the center is found. Once it is found, the next pattern is presented and this continues until the correct locations have been found for all patterns. Finding the location for each of the patterns in the set is classified as one trial. In the current study, participants were required to learn sets of 2, 4, 6, 8 and 10-pattern-location associations and were allowed six learning trials to learn each set.

### Procedure

Children were tested in a quiet room at their school in either single or two 20 –minute sessions (depending on the time taken to complete the associate learning tasks). Children were assessed in a single session, except when scheduled class breaks (recess, lunch), bathroom breaks, or signs of fatigue necessitated another testing session to complete the task on the same day. In this instance, the second session was approximately 20 minutes for re-familiarization with the experimenter and the testing environment. Children who attended a second testing session were given a practice trial on an alternative version of the task prior to resumption of testing. Initial training involved a verbal description of the task goal and a demonstration of rules on a three-pattern-location association version of the CPAL task. In the demonstration, probes for incorrect move types were used to examine children's understanding (e.g., once a location had been searched already the examiner asked “is the pattern in here?” while pointing to the previously searched location). Children completed two practice trials on the three-pattern-location association version of the CPAL to ensure that they understood the rules before proceeding to the test phase. Pilot work with five six-year-old children indicated that this provided sufficient practice for children to understand the task goal as determined by observations of performance. During the practice session, the examiner corrected all errors verbally on the first trial but not on the second practice trial. This included a statement of why any choice was incorrect (e.g., “not in there,” “the other pattern is in there already,” “you already looked there,” “there is nothing in there”). During testing, children completed the one exposure and six learning trials on the 2-, 4-, 6-, 8- and 10-pattern-location association versions in ascending order. An ascending, rather than a random order, was used so that testing could be ceased if difficulty prevented a child from completing the task within 20 minutes. The 20-minute time limit was identified from pilot testing and from previous studies of computer tests in young children [58]. In order to minimize potential discomfort for children the total time allowed for testing on the CPAL was 20 minutes and once this interval was reached the task finished automatically. Data from tests not completed within 20 minute was excluded from the analysis.

### Data Analysis

Performance on the CPAL was defined using the number of memory errors and the number of executive errors made while completing the six trials at each memory load. The error types classified as memory errors and executive errors are defined in Table 1. In addition to these a total error score was also computed. Thus, each participant provided a memory error, executive error and total error score for the 2-, 4-, 6-, 8- and 10-pattern-location association version of the CPAL. The main outcome measures were analyzed in three stages.

First, to evaluate whether memory and executive errors represented two separate dimensions of CPAL performance reflecting learning and executive function, we conducted confirmatory factor analyses of error types made on the 10-pattern location association version of the CPAL using Mplus version 7.11. In these analyses, we compared a 1-factor model with all error types (i.e., exploratory, distractor-search, between-search, within-search, and perseverative errors) loading on a single factor to a 2-factor model with these error types loading on two separate factors: memory errors (i.e., exploratory and distractor-search errors); and executive errors (i.e., between-search, within-search, and perseverative errors). Given the nested factor structures of these two models, a χ^2^ difference test was computed to compare their relative fit.

Second, total error scores for all age groups on the 2- and 4-pattern-location association versions of the CPAL showed substantial restriction of range. Therefore, for these levels, the numbers of memory and executive errors were not computed. Instead, median total error scores were computed for each age group and compared between age groups using Mann-Whitney U (MWU) tests.

Third, because data for the 6-, 8-, and 10-pattern-location association versions of the CPAL met assumptions necessary for parametric analysis, a series of repeated-measures analyses of variance (ANOVAs) were used to examine the interaction between increasing memory load and increasing age for total errors and also for memory and executive errors. For each of these analyses, age group (5–6 year olds, 7–8 year olds, 9–10 year olds) was entered as a between-subjects factor, and the number of total, memory or executive errors for the 6-, 8-, and 10-pattern-location association versions of the CPAL was entered as the within-subjects factors.

## Results

Examination of completion rates indicated that all of the 7–8 and 9–10 year olds completed all versions of the CPAL (100% completion rate) and only one child in the 5–6 year old group was unable to complete the 6-, 8-, and 10-pattern-location association versions of the CPAL (96.7% completion rate) within the allotted time. This data was excluded from the analysis. Table 2 provides descriptive statistics (means, standard deviations, range) for total error scores for all pattern-location association versions of the CPAL for the three age groups.

**Table 2:** Means, Standard Deviations, and Ranges for Total Errors on the CPAL2 – CPAL10.

Memory Load	Age Group (in years)					
	5–6 (n = 30)		7–8 (n = 49)		9–10 (n = 46)	
	Mean, SD	Range	Mean, SD	Range	Mean, SD	Range
2-assoc	5.5, 6.8	0–26	2.1, 2.9	0–17	0.8, 1.1	0–5
4-assoc	13.4, 12.0	0–50	8.1, 7.5	0–37	3.6, 4.5	0–21
6-assoc	34.5, 19.9	7–76	26.8. 18.8	4–73	19.9, 16.6	2–88
8-assoc	73.9, 38.0	0–168	60.5, 29.1	15–143	47.0, 21.9	17–101
10-assoc	102.6, 63.7	0–237	89.7, 52.8	7–219	68.9, 46.2	5–187

*Note. Assoc = association. CPAL2 = Continuous Paired Associate Learning Test-2 pattern-location version; CPAL10 = Continuous Paired Associate Learning Test-10 pattern-location version.*

To evaluate whether memory and executive errors represented two separate dimensions of CPAL performance reflecting learning and executive function, we conducted confirmatory factor analyses of error types made on the 10-pattern location association version of the CPAL using Mplus version 7.11. Because the vast majority (n = 114, 91.2%) of children did not make any perseverative errors on this version of the CPAL, these error types were excluded from these analyses. In these analyses, we compared a 1-factor model with all error types (i.e., exploratory, distractor-search, between-search, and within-search errors) loading on a single factor to a 2-factor model with these error types loading on two separate factors: memory errors (i.e., exploratory and distractor-search errors); and executive errors (i.e., between-search and within-search errors). Conventional fit statistics, including χ^2^, Akaike Information Criterion (AIC), Bayesian Information Criterion (BIC), Comparative Fit Index (CFI), Tucker-Lewis Index (TLI), and Root Mean Square Error of Approximation (RMSEA) were used to compare the fit of these two models. Given the nested factor structures of these two models, a χ^2^ difference test was also computed to compare the relative fit of these models.

Confirmatory factor analyses revealed the following fit statistics for the 1-factor model: χ^2^(2) = 11.23, p = .004, AIC = 3621.31, BIC = 3655.25, CFI = .979, TLI = .938, and RMSEA(90%CI) = .192, .094–.308. Fit statistics for the 2-factor model were as follows:χ^2^(1) = 2.77, p = .10, AIC = 3614.79, BIC = 3651.56, CFI = .996, TLI = .976, and RMSEA(90%CI) = .119, .000–.296. Aχ^2^ difference test revealed the the 2-factor model fit the data significantly better than the 1-factor model, χ^2^(1) = 12.25, p = .0005. Standardized factor loadings were .873 for both legal and distractor errors (i.e., memory errors factor); and .898 and .876 for between-search and within-search errors, respectively (i.e., executive errors factor). Thus, these error types were analyzed separately in this study.

Table 3 shows median total error scores for the 2- and 4-pattern-location association versions of the CPAL for the three age groups. For the 2- and 4-pattern-location association versions of the CPAL, the 9–10 year olds made significantly fewer errors than the 7–8 year olds, who made significantly fewer errors than the 5–6 year olds. At the 2-pattern-location association version, 20% of the 5–6 year olds (n = 6) and 7–8 year olds (n = 10) were able to perform the task error free. Additionally, 48% of the 9–10 year olds (n = 22) were able to perform the 2-pattern-location association version error free. At the 4-pattern-location association version, the task was performed error free on 3% of 5–6 year olds (n = 1), 8% of the 7–8 year olds (n = 4) and 24% of the 9–10 year olds (n = 11).

**Table 3:** Median Scores and Interquartile Ranges for Total Errors on the CPAL2 & CPAL4.

Memory Load	Age Group (in years)			Test of Difference (MWU, p)		
	5–6 (n = 30)	7–8 (n = 49)	9–10 (n = 46)	5–6v.9–10	5–6v.7–8	7–8v.9–10
2-association	2.5 (1–10)	1.0 (1–3)	1.0 (0–1)	308, <.01	511, .02	725, <.01
4-association	10.0 (4–25)	6.0 (4–10)	2.0 (1–5)	267, <.01	531, .04	612, <.01

*Note. CPAL2 = Continuous Paired Associate Learning Test-2 pattern-location version; CPAL4 = Continuous Paired Associate Learning Test-4 pattern-location version. MWU = Mann-Whitney U tests.*

**Figure 3:**
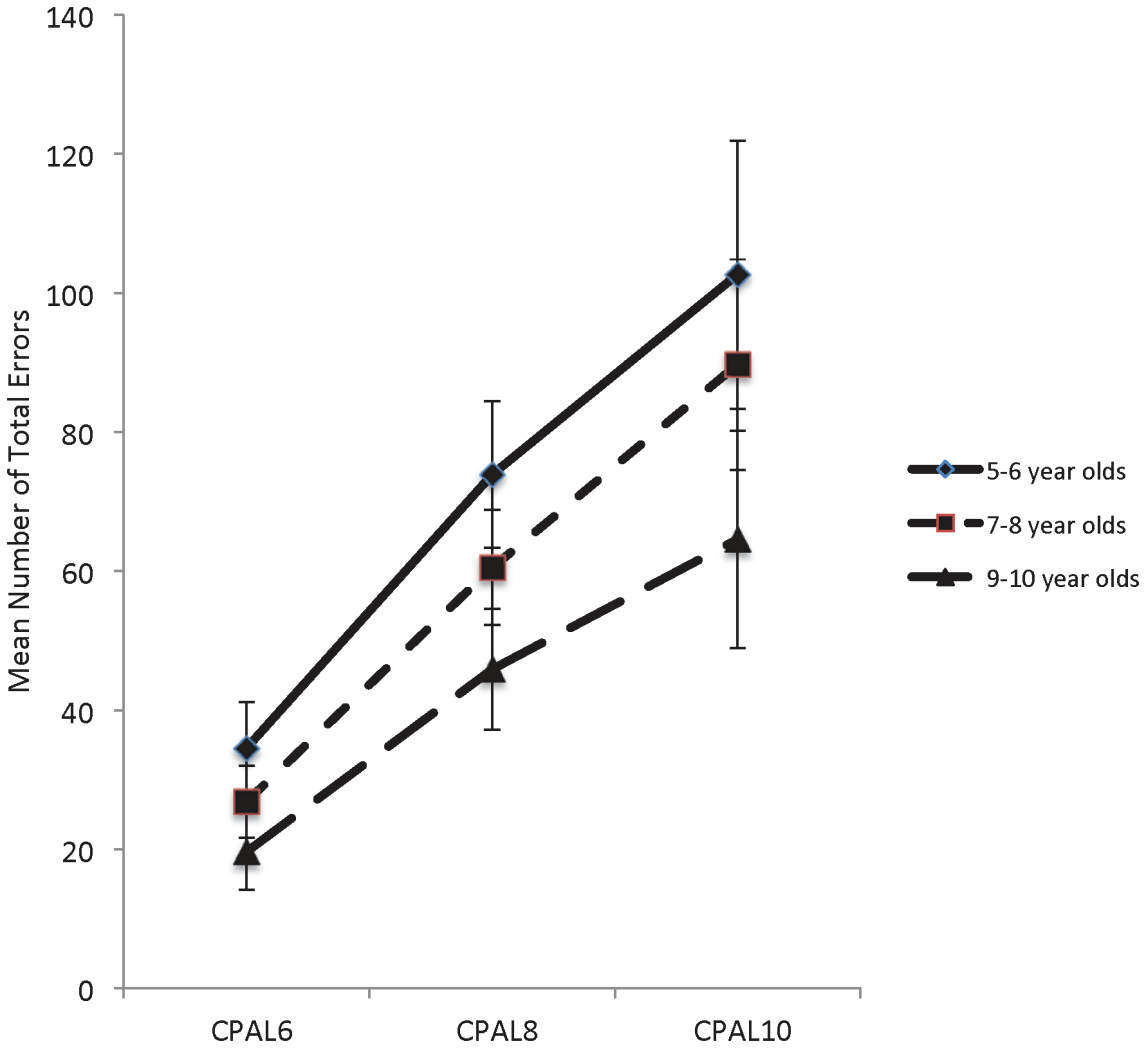
Figure 3a. Total errors (95% CI bars) made at 6, 8 and 10 memory loads in all age groups. Figure 3b. Memory errors (95% CI bars) made at 6, 8 and 10 memory loads in all age groups. Figure 3c. Executive errors (95% CI bars) made at 6, 8 and 10 memory loads in all age groups.

Figures 3a to 3c show group means and 95% confidence intervals for each of the three CPAL performance measures for the 6-, 8-, and 10-pattern-location association versions of the CPAL. For total errors (Figure 3a), repeated-measures ANOVA indicated significant main effects of age group (F(2,122) = 7.67, p = 0.001) and memory load (F(2,121) = 117.80, p<0.001) but the interaction of age group x memory load was not significant (F(2,122) = 2.26, p = 0.109). Further examination of the main effect of age for total errors indicated that 9–10 year olds made significantly fewer errors than 5–6 year olds (p = 0.001). There were no differences in total errors between 7–8 year olds and 9–10 year olds (p = 0.053) or 7–8 year olds and 5–6 year olds (p = 0.244). Further examination of the main effect of memory load for total errors indicated that performance worsened as memory load increased (6-pattern-location association <8-pattern-location association <10 pattern-location association; p<0.001 for all comparisons).

For memory errors (Figure 3b), repeated-measures ANOVA indicated significant main effects of age group (F(2,122) = 10.11, p<0.001) and memory load (F(2,121) = 75.21, p<0.001) but the interaction of age group x memory load was not significant (F(2,122) = 1.32, p = 0.270). Further examination of the main effect of age for memory errors indicated that 9–10 year olds made significantly fewer errors than 5–6 year olds (p<0.001) and 7–8 year olds (p = 0.009). There were no differences in memory errors between 7–8 year olds and 5–6 year olds (p = 0.270). Further examination of the main effect of memory load for memory errors indicated that performance worsened as memory load increased (6-pattern-location association <8-pattern-location association <10 pattern-location association; p<0.001 for all comparisons).

For executive errors (Figure 3c) repeated-measures ANOVA indicated significant main effects of age group (F(2,122) = 5.55, p = 0.005) and memory load (F(2,121) = 166.08, p<0.001), as well as a significant interaction of age group x memory load (F(2,122) = 3.16, p = 0.046). Further examination of the main effect of age for executive errors indicated that 9–10 year olds made significantly fewer errors than 5–6 year olds (p = 0.004). There were no differences in executive errors between 7–8 year olds and 9–10 year olds (p = 0.238) or 7–8 year olds and 5–6 year olds (p = 0.227). Further examination of the main effect of memory load for executive errors indicated that performance worsened as memory load increased (6-pattern-location association <8-pattern-location association <10 pattern-location association; p<0.001 for all comparisons). Further examination of the interaction for executive errors indicated that 5–6 year olds made significantly more errors as memory load increased (6-pattern-location association <8-pattern-location association <10 pattern-location association; p<0.05 for all comparisons). For 7–8 year olds, the same pattern was observed (6-pattern-location association <8-pattern-location association <10 pattern-location association; p<0.05 for all comparisons). Conversely, 9–10 year olds made significantly more errors as memory load increased from 6-pattern-location associations to 8-pattern-location associations; however, there was no statistically significant difference between 8-pattern-location associations and 10-pattern-location associations.

## Discussion

The results supported the first hypothesis that visual associate learning would become less efficient as memory load increased (Table 3 and Figure 3a). In children of all ages, the efficiency of associate learning decreased as memory load increased. However, at the lowest memory loads (2-pattern-location associations and 4-pattern-location associations), some children performed the task error free, suggesting that for those children the number of pattern-location associations did not exceed working memory capacity. While 20% (n = 6) of 5–6 year olds were able to perform the 2-pattern-location association version of the CPAL error free, only 3% (n = 1) were able to do so on the 4-pattern-location association version, suggesting that four chunks of information exceed working memory capacity for nearly all of the 5–6 year old children. Conversely, 24% (n = 11) of 9–10 year olds were able to perform the 4-pattern-location association version error free, indicating that older children typically had greater working memory capacity than younger children (see Table 3). As noted earlier, there is substantial variation in the working memory capacity measured using different tasks [48]. Nonetheless, our findings are largely consistent with previous work showing that working memory capacity increases with age and achieves approximately four items by 10 years of age [36]–[40], [59]. The notion that working memory capacity increases with age is not new and has been recognized by developmental theorists for over 40 years [60].

The results also supported the second hypothesis that, under conditions where working memory capacity was exceeded, visual associate learning would improve with increasing age. For CPAL conditions that required children to learn 6-, 8- or 10-pattern-location associations, 5–6 year old children made more errors than 7–8 year old children, who showed equivalent numbers of total errors as 9–10 year old children (Figure 3a). This finding is consistent with the age differences in visual associate learning reported previously [22], [24].

Examination of the memory and executive scores of the CPAL indicated that the memory and executive components of associate learning were affected differently by memory load under conditions where pattern-location associations exceed working memory capacity (6-pattern-location associations to 10-pattern-location associations). That memory and executive functions are necessary for associate learning is consistent with data from an extensive neuropsychological literature in both adults and children that shows performance on paired associate learning tasks to be impaired following focal disruption to frontal or medial temporal structures. For example, in adults with lesions of the frontal lobes [8]–[10], [32] and Parkinson's disease [34], [61], [62], as well as in children with ADHD [1], impaired performance has been interpreted to reflect difficulties in learning to select the appropriate response to a given stimulus from a set of stimuli [8]–[10] and strategic processing [1], [32], [34].

The results from this study showed that the efficiency of memory decreased under increasing memory load for memory errors and that the rate of decrease did not differ between the different age groups, though there were group differences in memory performance across all versions of the CPAL (Figures 3b). Conversely, analysis of executive errors indicated that younger children had greater difficulty than older children in their ability to use executive functions to optimize performance on the CPAL as memory load increased (Figure 3c). The nature of the errors (see Table 1 showing that within-search errors reflect failures to maintain the information in working memory capacity while between-search errors reflect failures in strategy use) contributing to the executive score indicate that working memory capacity continues to play a role in associate learning even after the number of pattern-location associations has exceeded capacity. The integration of working memory capacity and associate memory is consistent with Baddeley's influential multicomponent framework of working memory [41]–[43]. For Baddeley and colleagues, working memory refers to the ability to temporarily store and manipulate information in order to perform complex cognitive functions and is subserved by a set of interacting cognitive processes [43]. The framework consists of two subsidiary systems: a verbal store and a visuospatial store (phonological loop and visuospatial sketchpad), an attentional control system (the central executive), and a multidimensional buffer that integrates different sources of information from both within and outside of working memory (episodic buffer) [43].

In the process of searching for each of the patterns in the set (defined as a single trial), children need to retain in working memory (i.e., working memory capacity) the previous locations they searched during that trial while also attempting to recall the location for the pattern they are seeking from previous trials. As a result of this interaction between the episodic buffer and associate memory, improvement in performance on measures of associate learning as children age is also influenced by development of working memory capacity [63]. Taken together, these data suggest that as children age they are better able to employ strategy use and working memory to handle increasing memory loads. Conversely, the absence of an interaction between age group and memory load for memory errors suggest that memory processes do not change as a consequence of increasing memory load in older children versus younger children.

This is consistent with the results of other studies that have examined the development of memory and executive functions on separate tasks in children of the same age. These studies have concluded that performance on tasks of memory known to rely on the medial temporal lobes develops during early childhood [22], [24]. However, performance on tasks of executive functions, such as problem solving and strategy use, continues to develop through childhood and into adolescence [44]–[46]. Further evidence of the role of memory and executive functions in visual associate learning, as well as their differential developmental rates, was provided by modulating the difficulty level of a visual associate learning task (Nine Box Maze Test – Child Version; NBMT-CV) in children aged 5–12 years old [24]. The NBMT-CV included both an exposure trial during which the children were exposed to the to-be-remembered objects followed by learning trials of increasing task difficulty during which the children learned the object-location associations. At the lower difficulty level, no differences were noted between age groups. Conversely, 5–6 year olds consistently performed more poorly than the older children at the higher difficulty level. This was interpreted to indicate that memory was largely developed by 5–6 years old while differences in performance between age groups at the higher difficulty level were attributable to continued development of executive functions (i.e., strategy use).

In sum, the CPAL is a novel measure of visual paired associate learning that allows for the classification of different types of errors made during learning. This allows for the measurement of different cognitive processes, in particular memory and executive functions. Additionally, by varying the memory load of the task, it is also possible to obtain an estimate of working memory capacity. The CPAL is the first task that allows for the investigation of the different component processes of associate learning within the same task. Therefore, our study is the first to provide an understanding of how maturation in memory, executive functions and working memory capacity operate to form the foundation for maturation in visual paired associate learning. These data also provide neuropsychologists and psychologists with new information that can assist with their interpretation of poor visual associate learning in school aged children.

Several limitations of this study should be acknowledged. First, although the current data indicate that changes in performance on visual paired associate learning tasks through early to middle childhood are influenced by cognitive processes (i.e., memory and executive functions) that have different developmental trajectories, a prospective study is needed to fully elucidate the developmental course of these cognitive processes. Second, in order to establish the validity of the CPAL as a measure of associate learning in children, it is necessary to examine the extent to which well-validated neuropsychological measures of memory correlate with performance on the CPAL, as well as how children with memory disorders perform on this task. Studies examining performance on the CPAL in older adults with MCI or AD [49], [50] and in healthy adults challenged with psychopharmacological agents [51] currently provide evidence that this task is a sensitive measure of memory impairment. In addition, the current data provide a framework for considering the CPAL as a useful neuropsychological measure of visual associate learning in children. Despite these limitations, the current data suggest that the CPAL is a developmentally appropriate measure of visual associate learning in children as young as five years of age.
